# A modern day perspective on smoking in peripheral artery disease

**DOI:** 10.3389/fcvm.2023.1154708

**Published:** 2023-04-28

**Authors:** Leili Behrooz, Abdelrhman Abumoawad, Syed Husain M. Rizvi, Naomi M. Hamburg

**Affiliations:** ^1^ Whitaker Cardiovascular Institute, Boston University Chobanian and Avedisian School of Medicine, Section of Vascular Biology, Boston Medical Center, Boston, MA, United States; ^2^ Evans Department of Medicine and Whitaker Cardiovascular Institute, Boston University Chobanian & Avedisian School of Medicine, Boston, MA, United States

**Keywords:** smoking, peripheral artery disease (PAD), atherosclerosis, smoking cessation, nicotine replacement therapy, mechanism of injury

## Abstract

Peripheral artery disease (PAD) is associated with increased risk of cardiovascular morbidity and mortality, poor functional status, and lower quality of life. Cigarette smoking is a major preventable risk factor for PAD and is strongly associated with a higher risk of disease progression, worse post-procedural outcomes, and increased healthcare utilization. The arterial narrowing due to atherosclerotic lesions in PAD leads to decreased perfusion to the limbs and can ultimately cause arterial obstruction and limb ischemia. Endothelial cell dysfunction, oxidative stress, inflammation, and arterial stiffness are among the key events during the development of atherogenesis. In this review, we discuss the benefits of smoking cessation among patients with PAD and the use of smoking cessation methods including pharmacological treatment. Given that smoking cessation interventions remain underutilized, we highlight the importance of incorporating smoking cessation treatments as part of the medical management of patients with PAD. Regulatory approaches to reduce the uptake of tobacco product use and support smoking cessation have the potential to reduce the burden of PAD.

## 1. Introduction

Peripheral artery disease (PAD) is a major cardiovascular disease that affects more than 200 million people worldwide and an estimated 8.5 million people in the U.S (1–3). It is well known that patients with PAD have a higher risk of mortality and major cardiovascular events (4). Also, PAD is associated with the lowest quality of life among all symptomatic CVDs (5). Smoking is a modifiable risk factor for PAD, carrying a three to four-fold increased risk for the development of PAD (2, 4, 6).

Smoking contributes to the clinical expression of PAD and smoking cessation has clear clinical benefits. Novel tobacco products including electronic cigarettes (e-cigarettes) raise important questions about their value in reducing the harms of smoking compared to their risk of increasing smoking prevalence. Regulatory strategies that reduce tobacco use and enhance access to smoking cessation treatments have the potential to reduce the PAD burden. The current review focuses on the intersection of smoking with PAD and modern strategies to promote a tobacco-free future.

## 2. The burden of smoking and PAD

Although the population of adult smokers in the U.S. has declined, 12.5% of adults in the U.S. still smoke (7). Overall, the burden of smoking-related PAD deaths has declined modestly in both men and women over the past three decades due to the intense regulatory and public education efforts. Yet, in 2019, nearly a quarter of PAD-related deaths remain attributable to tobacco use globally (8). Secondhand smoke exposure confirmed by urinary cotinine levels has also been linked to clinical PAD (9). The Global Reduction of Atherothrombosis for Continued Health (REACH) Registry reported that 22% of patients with clinical PAD are current smokers, a prevalence that is double that of patients with other forms of atherosclerotic disease (5, 6). Although, PAD is three times more likely to develop in black patients compared to non-Hispanic white patients, prior studies have provided limited information about the association of smoking and incident PAD by race (10, 11). The Jackson Heart Study assessed the effect of smoking on Black patients with PAD and reported that smoking was associated with 2.2 times the probability of subclinical PAD measured by ankle-brachial index (ABI). Also, Black patients who were smoking more than >20 cigarettes per day and those with higher pack/year had significantly higher odds of subclinical PAD compared to those who were smoking <20 cigarettes per day confirming the importance of smoking intensity (11).

Active tobacco use in patients with PAD is strongly associated with a higher risk of disease progression, worse post-procedural outcomes, early failure of revascularization therapies, increased hospitalizations, and cardiovascular events including myocardial infarction and death (2, 12). Among patients with PAD who have undergone lower extremity revascularization, those who smoke more than one pack a day have a higher risk of major adverse limb events as well as amputation incidence at 1-year follow-up compared to those who smoked less. Patients who were current smokers had a higher mortality rate as well as a lower survival rate without amputation compared to patients who had quit smoking (13). Patients with PAD have poor functional status and quality of life. The PORTRAIT (Patient-Centered Outcomes Related to Treatment Practices in Peripheral Arterial Disease: Investigating Trajectories) trial reported that older age, female sex, poor access to care, and economic burden were among the risk factors associated with worse outcomes in patients with PAD (14). Current smokers had evidence of lower ABI and more proximal disease despite being younger.

## 3. Mechanisms of vascular injury due to smoking

**Figure 1:**
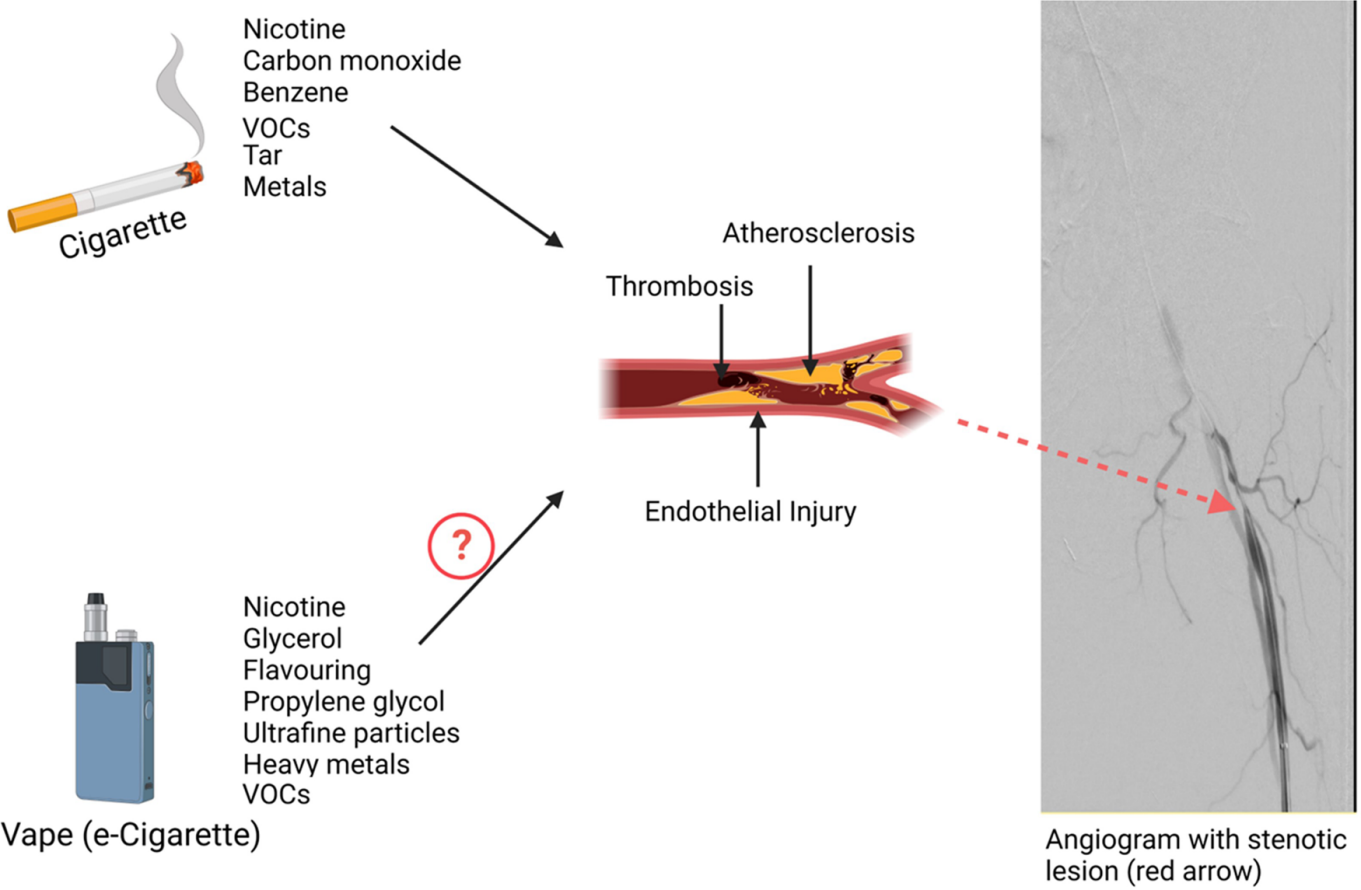
Impact of tobacco products on Peripheral Artery Disease (PAD). Combustible cigarettes release multiple potentially harmful constituents into tobacco smoke that induce endothelial injury, promote thrombosis, and accelerate atherothrombosis. Together the toxins in tobacco smoke adversely influence the peripheral vasculature leading to heightened PAD incidence and worse outcomes with established PAD.

Patients with PAD have disrupted blood flow to the limbs due to the formation of atherosclerotic plaque and the development of atherothrombosis. Cigarette smoking is a well-known contributor to the development of atherosclerosis and PAD (Figure 1). The interaction of genetic predisposition, harmful compounds released in cigarette smoke, metabolism of toxins, and intersection with concomitant risk factors influence the individual PAD expression (15, 16). Even a low intensity of ongoing smoking increases cardiovascular risk suggesting that low-intensity toxin exposure not just cumulative lifetime exposure contributes to vascular disease.

The pathophysiology of PAD is complex and multifactorial and involves a variety of cells including endothelial cells, inflammatory cells, vascular smooth muscle cells, and platelets (17). There is evidence from preclinical models linking multiple components of cigarette smoke to the process of injury in all these cell types. Cigarette smoke exposure causes oxidative stress and endothelial cell dysfunction reducing nitric oxide (NO) bioavailability which aggregates endothelial leukocyte interaction, induces the production of inflammatory and proatherogenic cytokines, impairs smooth muscle cell function, and amplifies platelet activation which leads to atherothrombosis (16, 18).

Multiple components of cigarette smoke are potentially harmful compounds. There are many chemicals produced in cigarette smoke that might be responsible for vascular injury. Nicotine is one of the chemicals in cigarette smoke among more than 7,000 different chemicals (19–21). Though nicotine replacement therapy (NRT) is generally safe, there is evidence that links nicotine to adverse cardiovascular effects (22–24). Nicotine causes vasoconstriction through multiple mechanisms such as stimulating the endothelial cells' alpha-adrenergic receptors by catecholamines and reducing the availability of NO in the endothelial cells (25). Nicotine inhibits GTP cyclohydrolase 1 (GTPCH1), the key enzyme in B tetrahydrobiopterin (BH4) synthesis. BH4 is necessary for the endothelial NO synthase (eNOS) to produce NO. Thus, in theabsence of BH4, eNOS synthesizes superoxide instead of NO (26, 27). Nicotine has been shown to promote atherogenesis in animal models and contribute to the acute cardiovascular effects of e-cigarettes (28, 29). Thus, it will be important to investigate the impact of reduced nicotine tobacco products and consider reducing nicotine content in all tobacco products.

There are additional harmful compounds that are important in the process of atherosclerosis and the development of PAD. Cigarette smoke includes volatile organic compounds (VOCs) such as aldehydes, which are classified as saturated aldehydes including formaldehyde and acetaldehyde, and unsaturated aldehydes acrolein and crotonaldehyde. Acrolein for example can accelerate atherosclerosis through elevated levels of oxidative stress and platelet activation and suppression of circulating angiogenic cell levels (30, 31).

E-cigarettes are devices that heat a liquid comprised of propylene glycol, glycerin, and typically nicotine and flavors to form an aerosol. The aerosol contains fewer toxins than tobacco smoke but has substantial levels of VOCs. Ogunwale et al. demonstrated that some aldehydes such as acrolein, formaldehyde, and acetaldehyde are also present in e-cigarette aerosols (32). E-cigarette use can instigate oxidative stress and inflammation leading to endothelial dysfunction, a major contributor to atherosclerosis. It has been shown that acute use of e-cigarettes induces vascular dysfunction similar to tobacco smoking (33–35). Mohammadi et al. demonstrated that both e-cigarette users and cigarette smokers had lower flow-mediated dilation, reduced vascular endothelial growth factor, and NO secretion compared to non-users (36). Studies have evaluated the effect of e-cigarettes on endothelial cell function by examining freshly isolated venous endothelial cells of e-cigarette users. They reported that e-cigarettes were associated with impaired eNOS activation and endothelial cell dysfunction. While some studies have related the association of e-cigarettes and endothelial cell dysfunction to nicotine, other studies have reported that e-cigarettes induce endothelial cell dysfunction independent of nicotine (37, 38). It is important to note that Flavoring compounds were also found injurious to endothelial cells and they continue to be included in the majority of e-liquids (39, 40). Thus, e-cigarette aerosol has the potential to induce adverse cardiovascular outcomes, though the relative impact of e-cigarette use compared to cigarette smoking on clinical events remains poorly understood.

## 4. Benefits of smoking cessation in PAD

**Figure 2:**
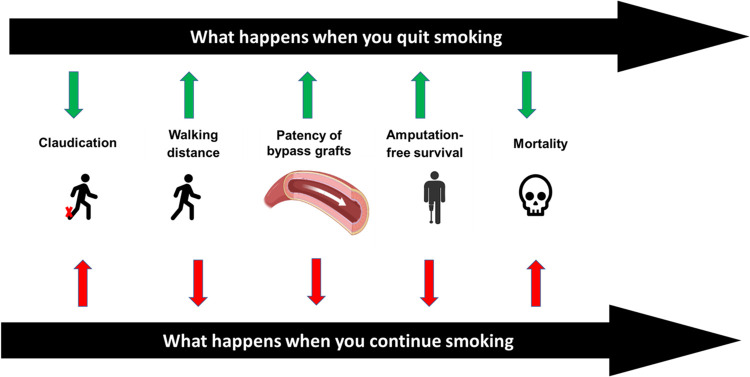
Benefits of smoking cessation on clinical outcomes in patients with PAD.

Four out of every five patients with PAD are either current or former smokers (41). According to the REACH registry, 22% of patients with clinically evident PAD continue to use tobacco products (6). There is a rapid decline in cardiovascular risk and major adverse cardiovascular events within a few years after smoking cessation (2, 42). Similarly patients with PAD have better outcomes with smoking cessation across multiple clinical dimensions (Figure 2).

### 4.1. Claudication and walking distance

Smoking is a known independent risk factor for claudication (41, 43). Hughson et al. demonstrated that male and female smokers are more likely to develop intermittent claudication by fifteen and seven times more compared with non-smoker males and females, respectively (44). Smoking cessation has the potential to improve intermittent claudication. In a study by Quick et al. looking into ankle pressures, intermittent claudication, and exercise tolerance, the cohort of patients with PAD who continued to smoke showed a significant decrease in ankle pressures and had no change in their maximum treadmill walking distance. However, the cohort that quit smoking showed significant improvement in the maximum treadmill walking distance over a mean period of 10 months (45).

### 4.2. Graft failure and patency

Multiple studies have shown the negative impact of smoking on the patency of bypass grafts for PAD (46, 47). A meta-analysis of 29 studies looking at the relationship between smoking and the patency of lower extremity bypass grafts, reported that smoking after lower limb bypass surgery results in a threefold increased risk of graft failure and a number needed to harm of only four. A clear dose-response relationship was present, with decreased patency in heavy smokers compared with moderate smokers. It was also reported that smoking cessation restored patency rates towards the never-smokers group (46).

### 4.3. Mortality and amputation-free survival

The mortality rate among patients with PAD and critical limb ischemia (CLI) is higher compared to the general population. One of every three patients enrolled in the Best Endovascular vs. Best Surgical Therapy in Patients With Critical Limb Ischemia (BEST-CLI) trial died within three years (48). Armstrong et al. examined the association of smoking cessation with mortality and amputation-free survival. Among a retrospective cohort of 739 patients with claudication or CLI using the PAD-University of California, Davis Registry; mortality was lower in the successful smoking quitters cohort compared with the continued smokers cohort (14% vs. 31% at five years). The mortality benefit was more evident in the cohort of patients with CLI (18% vs. 43%, respectively) (49). Also, improved amputation-free survival was noted in the successful smoking quitters cohort compared with the continued smoker cohort with a hazard ratio of 0.43 (95% CI, 0.22–0.86). A recent study by Reitz et al. assessed 14,350 patients with intermittent claudication who underwent revascularization between 2011 and 2019, from the Veterans Affairs Surgical Quality Improvement Program. They reported that the 30-day mortality was higher (0.6% vs. 0.1%) among the smoker cohort regardless of the procedure modality: endovascular, open, or hybrid revascularization. Also, graft failure was higher across the smoker cohort (2.2% vs. 0.7%) (50, 51).

## 5. Economic burden of smoking on PAD

Despite the decline in smoking rates, healthcare costs related to tobacco use are staggering (52). Analysis of data from the REACH registry estimated the total cost of vascular-related hospitalizations was $21 billion in the U.S. in 2004, with most costs associated with revascularization procedures (53). A retrospective, cross-sectional study using data from 22,202 patients with PAD, found that hospitalization rates were higher for smokers compared to non-smokers. Smokers had significantly higher rates of more than one hospitalization within a 1-year period compared to non-smokers (49% vs. 36.4%, respectively). The primary hospitalization diagnosis for smokers was significantly more likely related to atherosclerotic disease compared to non-smokers. Smoking was associated with a 35% higher annual hospitalization rate and $18,000 higher annual cost per patient in patients with PAD who smoked (12). High healthcare costs in addition to worse clinical outcomes associated with patients with PAD who smoke highlight the importance of effective measures for smoking cessation to halt the progression of the disease and reduce the economic burden of PAD.

## 6. Underutilization of smoking cessation treatment

The American Heart Association/American College of Cardiology 2016 guidelines for the management of patients with PAD who smoke recommend using pharmacotherapy and/or referral to a smoking cessation program (54). Data from the national health interview surveys reported that 68% of adult smokers wanted to quit and more than half of adult cigarette smokers (55%) have made a quit attempt but only 7.5% ofsmokers were successful (55, 56). A randomized clinical trial reported that patients with PAD with active smoking who received the intensive intervention (physician advice, smoking cessation counseling, and pharmacologic treatment) had 21.3% rate of 6-month abstinence as compared to 6.8% in the less intensive treatment group who only received verbal advice to quit smoking (57). Despite the evidence on the efficacy of these interventions, a relatively low number of patients with PAD who are smokers are referred for smoking cessation counseling or pharmacologic treatment (4). Kalbaugh et al. reported that smokers with PAD were only offered smoking cessation counseling or pharmacologic treatment in about one-third of visits (58). An analysis from the PORTRAIT registry by Patel et al. assessed the smoking rate and the smoking cessation interventions offered to patients with PAD who presented with new or worsening PAD symptoms and examined the changes in smoking behavior at 1-year follow-up. The predominant strategy used by providers for smoking cessation was to tell them to stop. Out of 474 patients with PAD who were active smokers, less than one in five were referred to a smoking cessation counseling program and only one in ten had received pharmacologic treatment. Among patients who quit smoking, more than a third relapsed which highlights the challenge of continuous abstinence from smoking for patients (4).

Incorporating smoking cessation interventions into the vascular clinical workflow has the potential efficacy for higher rates of smoking cessation. The Vascular Physician Offer and Report (VAPOR) trial assessed the feasibility and efficacy of standardized smoking cessation intervention for patients with PAD who were current smokers offered by vascular surgeons (59). Patients were randomized to either receive the “offer and report” smoking cessation intervention vs. usual care. The intervention included: (1) physician-delivered advice for smoking cessation, (2) NRT prescription, and (3) active referral for telephone-based smoking cessation. They reported that delivering smoking cessation intervention including NRT by vascular clinicians is feasible.

## 7. Modalities to support smoking cessation

**Figure 3:**
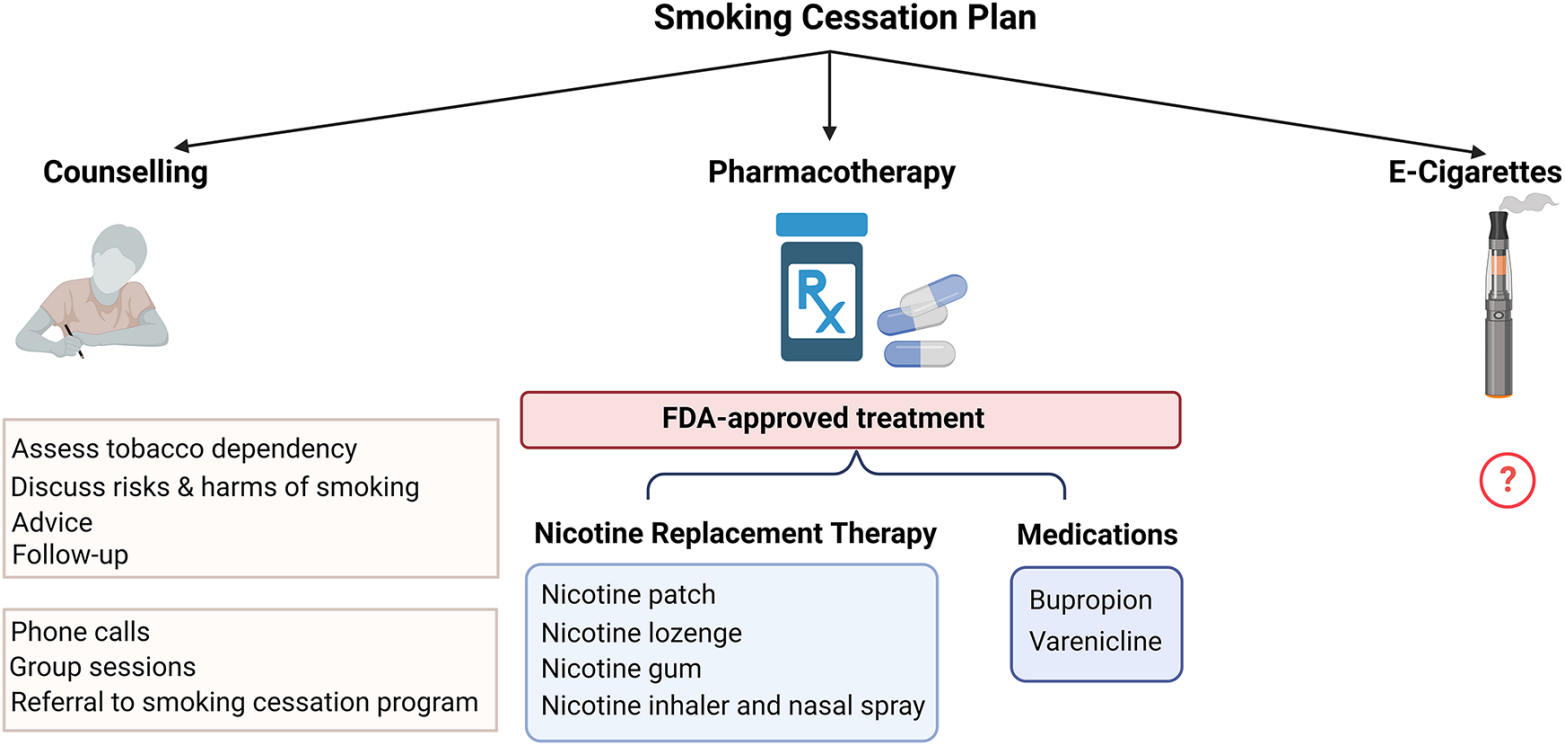
Approach to promoting smoking cessation in patients with PAD.

There are multiple approved methods that are used for smoking cessation which can either be used alone or in combination including pharmacologic therapy and behavioral approaches with smoking cessation counseling programs. E-cigarettes have been proposed as an aid for smoking cessation but there is a need for more information on their efficacy and safety (Figure 3).

### 7.1. Pharmacological therapies

U.S. Food and Drug Administration (FDA)-approved pharmacologic smoking cessation options include nicotine patches, nicotine lozenges, nicotine gum, nicotine inhaler, nicotine nasal spray, as well as medications including bupropion, and varenicline. NRT assists with smoking cessation by providing nicotine instead of cigarettes to suppress the urge to smoke and prevent nicotine withdrawal symptoms. It has been shown that treatment with dual NRT using a patch combined with either gum or lozenges is more effective than a single NRT due to delivering nicotine in both basal and bolus dosing. Though not tested in patients with PAD specifically, NRT has been shown to be safe in patients with established CVD (60). Bupropion enhances smoking cessation rates compared to placebo. Varenicline has been found to have the greatest success among all options with no reported neuropsychiatric adverse effects compared to placebo (61).

### 7.2. E-cigarettes

E-cigarettes have been introduced in the past two decades as an alternative product to combustible cigarettes and are frequently used in conjunction with or as a replacement for smoking cigarettes. E-cigarettes provide nicotine without burning tobacco and the aerosol has lower amounts of selected potentially harmful compounds (62). However, the presence of VOCs and flavorings still has the potential for cardiovascular injury. There is limited data regarding the health effects of e-cigarettes including CVD. A recent longitudinal study reported no significant difference in cardiovascular risk between exclusive e-cigarette users and non-users (not using cigarettes or e-cigarettes). However, dual use of e-cigarettes and combustible cigarettes was found to have an increased risk of CVD when compared to non-use. The study suggests that the health benefits of e-cigarettes are only apparent from the complete replacement of cigarettes with e-cigarettes but not from dual use with ongoing smoking (63). Future longitudinal research is needed to evaluate the long-term health effects of e-cigarettes on PAD outcomes.

In terms of efficacy for promoting smoking cessation, there remains limited data. A randomized trial compared the 1-year abstinence rate among smokers who received NRT products of their choice either alone or combined vs. smokers who received e-cigarettes. They reported that e-cigarettes were more effective with higher abstinence rates compared to NRT (64). A non-randomized prospective cohort study evaluated the effectiveness of e-cigarettes on smoking cessation compared to NRT. They reported that smokers who used e-cigarettes alone or in combination with NRT were more likely to report abstinence at 4–6 weeks follow-up compared to smokers using NRT alone (65). Importantly, patients in the e-cigarette group had a high rate of continued e-cigarette use. Thus, it will be crucial to understand the longitudinal impact of e-cigarettes on PAD outcomes.

### 7.3. Behavioral therapy (stepped approach)

The 2018 ACC Expert Consensus Decision Pathway on Tobacco Cessation recommends using both behavioral and pharmacologic therapy. Physicians taking care of patients who smoke should use a four steps plan to help with smoking cessation. First, patients should be asked about their smoking habits and their level of nicotine dependence which has been shown to correlate with their relapse rates after quit attempts. The next step is to give patients clear and personalized advice about the benefits of quitting. It is important that the information provided to patients is tailored toward their specific situation and personal health rather than general advice about the harms of smoking. The third step is to offer patients smoking cessation therapies including behavioral support and pharmacologic treatment. The use of pharmacotherapy is of critical importance and should be offered to every patient who smokes. The next step is to follow up with patients especially within the early period after they quit as the relapse rates are high during this time. Patients require continuous support and encouragement from their healthcare providers to remain smoke-free (61).

## 8. Regulatory approaches to reduce tobacco use

Moreover, it is important to develop policies and regulations at a global, government, and organizational level to help with smoking cessation and limit access to tobacco-related products. A joint opinion was issued by the American Heart Association, the World Health Foundation, the American College of Cardiology, and the European Society of Cardiology to decrease and eradicate tobacco use. Suggested strategies include increasing the price and taxation of tobacco products, eliminating advertisements for tobacco products, banning smoking in indoor spaces, having smoke-free workplaces, policies to protect people from secondhand smoke exposure, and characterizing flavors from tobacco products. Two states in the U.S. have banned all flavored tobacco products and it will be important to collect information regarding the effects on both youth and established tobacco product users. Another approach proposed by the FDA is to reduce nicotine content in combustible cigarettes. Additional scientific evidence regarding the specific harmful toxins in tobacco products and how these compounds impact cardiovascular health is crucial to support strong regulatory policies. To address the global tobacco epidemic, we need to advocate for policies proven to limit tobacco use and for the government to take stronger actions to protect public health (66).

## 9. Future directions

The present review emphasizes the significant harmful impact of smoking on patients with PAD. Worse clinical outcomes and the high healthcare costs associated with PAD highlight the importance of smoking cessation for this population. Although many smokers with PAD want to quit, the overall success rates for smoking cessation among this patient population are underwhelming. There is clear underutilization of formal smoking cessation interventions for smokers with PAD. To tackle this issue, we need more effective strategies to help patients quit and also provide ongoing support for continuous abstinence from smoking. More research is needed to assess the long-term effects of smoking cessation interventions including counseling, pharmacotherapy, and the efficacy of e-cigarettes. Also, it is important to advocate for implementing regulatory policies and public health approaches to limit access to tobacco products.
